# Changing patterns in global blindness: 1988–2008

**Published:** 2008-09

**Authors:** Allen Foster, Clare Gilbert, Gordon Johnson

**Affiliations:** Professor, International Centre for Eye Health, London School of Hygiene and Tropical Medicine, Keppel Street, London WC1E 7HT, UK.; Professor, International Centre for Eye Health; Medical Advisor, Sightsavers International, UK.; Honorary Professor, International Centre for Eye Health; Emeritus Professor of Preventive Ophthalmology, Institute of Ophthalmology, University College London, UK.

**Figure:**
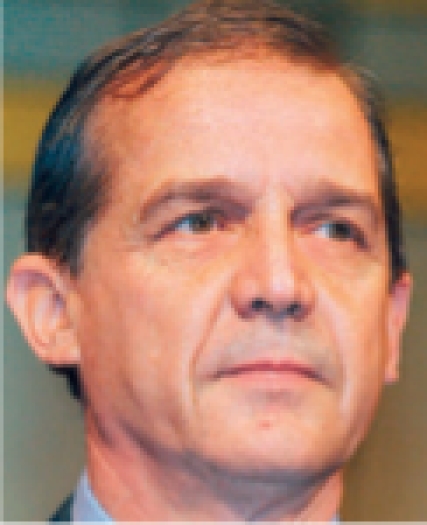


**Figure:**
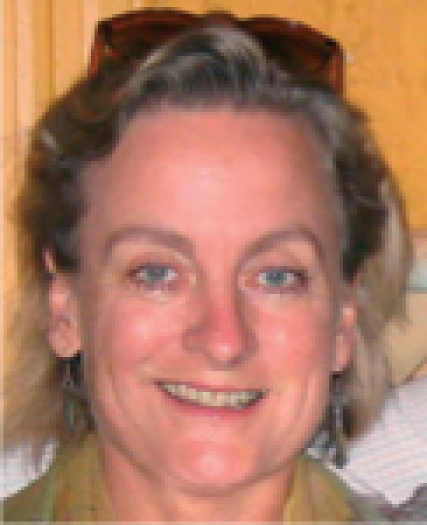


**Figure:**
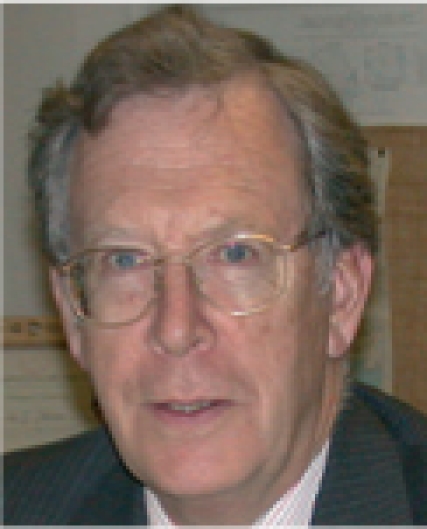


## Prevalence of visual impairment

### Changing demography

When the *Community Eye Health Journal* was launched in 1988, the world population was approximately 5.1 billion. Over the last 20 years, it has increased by approximately 30%, reaching 6.7 billion in 2008. During the same period, the world population has also become proportionally older, as the number of people aged 65 years and over has increased by approximately 55%, from 320 million in 1988 to 500 million in 2008. Since the prevalence of visual impairment becomes higher as people age, this combination of an increasing population and an ageing population is expected to cause a significant increase in the total number of blind people.^1^

### Estimates of the number of people with visual impairment worldwide

In 1988, the number of people who were blind (visual acuity (VA) < 3/60 in the better eye) was estimated to be 37 million worldwide. By 2002–04, the latest period for which we have data (see Table 1), it was estimated to be 45 million: 8 million blind due to uncorrected refractive error and 37 million blind due to other causes.^2^^,^^3^ It is thought that at least 60% of blind people are women.

**Table 1:** ***Most recent estimates of the number of people with visual impairment (blindness and low vision) worldwide^2^^,^^3^^,^^4^***

Definition		Number of people (millions)
Blindness (eye disease)	<33/60 to no light perception	37
Blindness (refractive error)	<33/60 to light perception	8
**Blindness (all causes)**		**45**
Low vision (eye disease)	<6/18 to 3/60	124
Low vision (refractive error)	<6/18 to 3/60	145
**Low vision (all causes)**		**269**
**Total: Visual impairment (all causes)**		**314**

**Figure:**
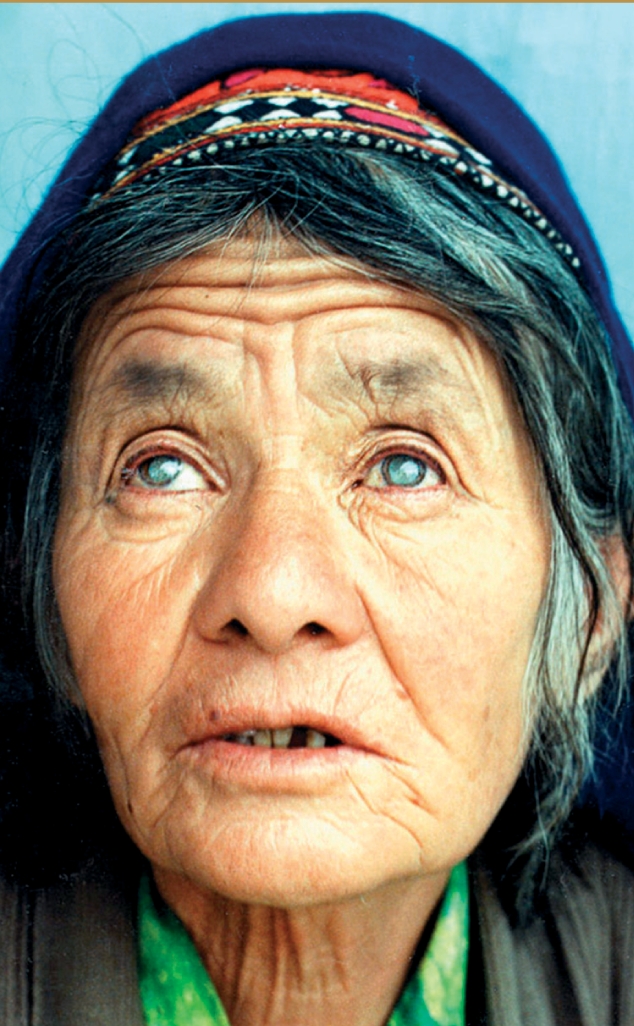
Cataract has remained the major cause of blindness worldwide for the past 20 years

Little was known in 1988 about the prevalence of low vision (VA <6/18 to 3/60). In 2002, the number of people with low vision was estimated to be 124 million worldwide, but this excluded low vision due to refractive error.^2^ Owing to a lack of data from surveys, it has only very recently become possible to estimate that there are 145 million people with low vision due to refractive error.^3^ This figure brings the overall number of people with low vision to 269 million.

In total, the number of people with visual impairment (which includes both low vision and blindness) is therefore estimated to be 314 million worldwide.

## Causes of blindness

**Figure 1:**
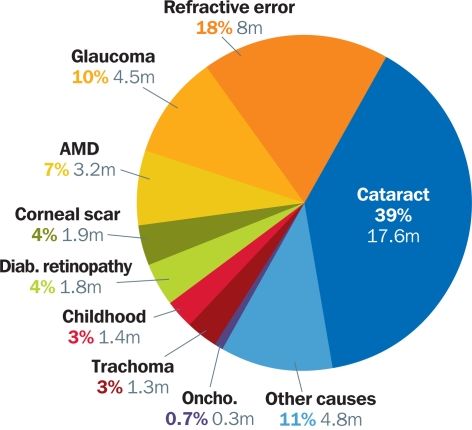
Proportion of cases of blindness due to each major cause∗

Over the last twenty years, the causes of blindness have changed in proportion and actual number. Cataract has remained the major cause of blindness globally. It is particularly important in Asia. The numbers of people blinded by trachoma, onchocerciasis, and vitamin A deficiency have tended to decrease over the last twenty years. This is due to improvements in nutrition, water supplies, sanitation, and measles immunisation coverage, as well as to the provision of certain therapeutic medicines: ivermectin (Mectizan®), vitamin A, and antibiotics. Figure 1 shows the proportion of cases of blindness due to each major cause, according to the most recent estimates.

### Cataract

Over the past twenty years, the major advance in the treatment of cataract has been the worldwide availability of low-cost, good-quality intraocular lenses (IOLs) since the early 1990s. Their routine use has resulted in an increase in both the quality of visual outcome for patients (as shown by population-based rapid assessment of cataract surgical services)^5^ and the willingness of surgeons to perform cataract surgery at an earlier time, before blindness has developed. This is detailed in the article on page 40. There is evidence that, since the introduction of IOLs, there has been an increase in cataract surgical rates around the world, and particularly in low-income countries.^4^

Two other important developments in the past twenty years have been the popularisation of phacoemulsification and the introduction of small incision cataract surgery (SICS). Both have resulted in a faster and better restoration of visual acuity. With SICS, in addition, the cost per operation is also lower.

Although it is difficult to obtain accurate figures, it is likely that the global number of cataract operations has increased from about 5 million per year in 1988 to around 15 million per year now.

Despite these overall positive developments, we should not be complacent: 17 million people are blind today because they have not yet received cataract surgery.

### Trachoma

In 1988, it was estimated that 150 million children were infected; this number had fallen to approximately 84 million by 2004.^6^ Similarly, the number of people blind from trachoma decreased from approximately 5 million in 1988 to 1.3 million in 2002.

The SAFE strategy for trachoma control has become widely accepted, tarsal rotation has been shown to be the preferred surgical procedure for trichiasis, and oral azithromycin has become the first-choice antibiotic for mass treatment of communities with endemic trachoma infection (as shown in the article on page 43). It is also highly likely that improvements in water supply and sanitation have significantly reduced the transmission of trachoma infection in poor rural communities in Africa and Asia. However, more investigative work is required in order to reduce recurrence after trichiasis surgery and to identify the most cost-effective strategies for the distribution of azithromycin.

### Onchocerciasis

In 1988, onchocerciasis was a significant cause of blindness in many countries in Africa. This same year, however, saw important developments in the treatment of the disease: Merck & Co. had registered the microfilaricide ivermectin (Mectizan®) a year earlier and its Mectizan® Donation Programme came into effect, providing Mectizan® free of charge to individuals and communities with onchocerciasis, as shown in the article on page 43. Twenty years on, the severity of onchocerciasis infection is decreasing and the number of people developing vision loss has markedly decreased. The figures for 2007 indicate that over 50 million people are now receiving Mectizan® on an annual basis through community-directed treatment programmes.

### Childhood blindness

Although vitamin A deficiency was a well-recognised cause of blindness in children twenty years ago, little work had been done up to that time on the magnitude and causes of childhood blindness. The article on page 46 presents an overview of the data collected and the lessons learnt over the past twenty years.

These data show marked variations according to the socio-economic status of the community. For example, vitamin A deficiency still occurs in children under five years old living in very poor families and, today, rising food prices worldwide may aggravate this situation further. Similarly, retinopathy of prematurity has emerged as a significant problem in middle-income countries and in urban centres of the developing world. The most important treatable cause of childhood blindness, however, remains untreated or poorly treated cataract, which is responsible for 5–20% of all cases.

### Refractive error

Little was known in 1988 about the magnitude of visual loss due to refractive error. This was due to the fact that the World Health Organization's (WHO) definition of blindness excluded correctable refractive error, which was therefore not recorded in surveys. Since then, some population-based blindness surveys have included people who cannot see because they have no spectacles and specific surveys have been done to assess refractive error in school children. Figures published in 2008 indicate that, due to uncorrected refractive error, there are 145 million people with VA ranging from <6/18 to 3/60 and 8 million people who are blind (VA <3/60) (see Table 1).^3^ Spectacles have generally become more available and more affordable, but in many countries there is still a need for good refraction services and for appropriate dispensing of low-cost but good-quality spectacles.

**Figure:**
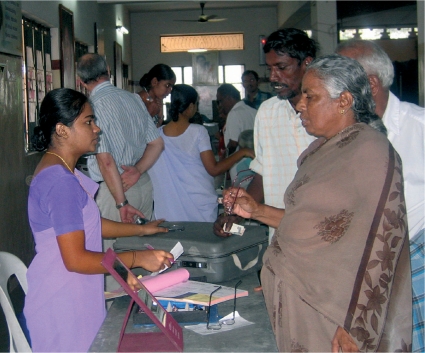
A patient collects her spectacles. INDIA

### Glaucoma

During the last twenty years, work has been undertaken to develop improved definitions and classifications of glaucoma. This has allowed for better estimates to be made of the number of people with this condition.^7^ It is likely that the current global estimate of 4.5 million people blind due to glaucoma actually falls short of the true figure, as many surveys do not include an assessment of visual field loss and are limited to a definition of blindness based only on visual acuity. Globally, 60 million people are likely to have one of the glaucomas and up to 8 million may be blind because of this disease.

Because no simple, specific, and sensitive test exists for this condition, population-based screening cannot at present be advocated; opportunistic case detection should, however, be encouraged. Unfortunately, in many low- and middle-income countries, effective treatment for glaucoma is still out of reach: medical treatment requires the availability of affordable drugs and long-term patient compliance; surgical treatment requires patient acceptance, as well as surgical skill, experience, and the capacity for long-term follow-up. This is difficult to achieve in some settings.

### Diabetic retinopathy

In 1988, there were no data on the global prevalence of diabetic retinopathy or of blindness resulting from this condition. It is now estimated that there are approximately 171 million people with diabetes worldwide. Of these people, probably 10–20% have some form of retinopathy and around 1.78 million are blind. There are now better-defined screening procedures and agreed treatment protocols based upon evidence from clinical trials. In appropriate settings, therefore, there can now be a public health approach to the control of visual loss from diabetes.^8^

### Age-related macular degeneration (AMD)

As life expectancy increases, AMD is becoming a more important problem, not only in high-income, but also in middle-income countries (see article on page 48). In 2002, it was estimated that 3.2 million people were blind from AMD. As yet, there is no proven prevention for AMD although smoking has been shown to be an important risk factor. Various surgical procedures are being tried in selected cases and recent studies indicate that vascular endothelial growth factor (VEGF) blockers can delay or stop progression of vascular AMD (see article on page 50). In spite of promising recent developments, there is, however, no proven therapy to reverse the degenerative process in all cases and current therapies remain expensive.^9^

## Making a difference with VISION 2020: The Right to Sight

In 1988, the WHO Prevention of Blindness (PBL) programme and the International Agency for the Prevention of Blindness (IAPB) had been in existence for ten years. Over the next decade, several important developments made it possible to conceive of a global initiative to eliminate avoidable blindness: the Mectizan® Donation Programme was established in 1987, low-cost IOLs became available in the early 1990s, and the SAFE strategy was launched in 1996. In addition, the relationship between vitamin A deficiency and childhood mortality had already been documented.

Drawing on their experiences of cost-effective eye care delivery systems in several countries in the 1980s and 1990s, including in India and The Gambia, a group of nongovernmental development organisations (NGDOs), together with the WHO, launched VISION 2020: The Right to Sight in 1999. This is a global initiative to eliminate avoidable blindness from cataract, trachoma, onchocerciasis, refractive error, vitamin A deficiency, and other causes of blindness in children by the year 2020.

The World Health Assembly has since adopted resolutions urging its member states to adopt the VISION 2020 principles. More than 90 NGDOs, agencies, and institutions, together with a number of major corporations, are now working together in this global partnership.

There is little doubt that the VISION 2020 initiative has raised awareness concerning blindness and the cost-effectiveness of available interventions. It has mobilised both government and private funding for eye care and it has generated a global public-private partnership working with a clearly defined focus and strategy.

Estimates of global blindness made in 2002 were 15 million lower than the projections made for this same year when VISION 2020 was launched. There is also evidence that the number of people who are blind due to onchocerciasis and trachoma has decreased, as well as evidence of increasing cataract surgical rates in many countries. Our challenge now is to build on what has been achieved and to focus resources on the poorest communities in the world. The goal of VISION 2020 is to enable all persons to receive eye care and have the right to sight – which is one of their fundamental human rights.
